# Multivalent Aptamer/Gold Nanoparticle–Modified Graphene Oxide for Mass Spectrometry–Based Tumor Tissue Imaging

**DOI:** 10.1038/srep10292

**Published:** 2015-05-14

**Authors:** Rong-Cing Huang, Wei-Jane Chiu, Irving Po-Jung Lai, Chih-Ching Huang

**Affiliations:** 1 Department of Bioscience and Biotechnology, National Taiwan Ocean University, Keelung, 20224, Taiwan; 2 Center of Excellence for the Oceans, National Taiwan Ocean University, Keelung, 20224, Taiwan; 3 School of Pharmacy, College of Pharmacy, Kaohsiung Medical University, Kaohsiung, 80708, Taiwan

## Abstract

The protein mucin1 (MUC1) is an attractive target for cancer biomarkers because it is overexpressed in most adenocarcinomas. In this study, we exploited a MUC1-binding aptamer (Apt_MUC1_) as a targeting agent for nanoparticle-based imaging systems coupled with laser desorption/ionization mass spectrometry (LDI-MS). We found that Apt_MUC1_-conjugated gold nanoparticles immobilized, through hydrophobic and π–π interactions, on graphene oxide (Apt_MUC1_–Au NPs/GO) bound effectively to MUC1 units on tumor cell membranes. The ultrahigh density and high flexibility of Apt_MUC1_ on the GO surface enhanced the platform’s cooperative and multivalent binding affinity for MUC1 on cell membranes. After we had labeled MUC1-overexpressing MCF-7 cells (human breast adenocarcinoma cell line) with Apt_MUC1_–Au NPs/GO, we used LDI-MS to monitor Au cluster ions ([Au_*n*_]^+^; *n* = 1–3), resulting in the detection of as few as 100 MCF-7 cells. We also employed this Apt_MUC1_–Au NPs/GO–LDI-MS system to analyze four different MUC1 expression cell lines. In addition, the Apt_MUC1_–Au NPs/GO platform could be used further as a labeling agent for tumor tissue imaging when coupled with LDI-MS. Thus, Apt–Au NPs/GO can function as a highly amplified signal transducer through the formation of large Au clusters ions during LDI-MS analysis.

Biological imaging enables the morphological features of organs to be correlated with pathological symptoms; this process is important for finding biomarkers and subsequently using them for the diagnosis of diseases^1^. Among the different technologies used for tissue imaging, mass spectrometry (MS) for molecular imaging has attracted a great deal of interest for the chemical characterization of biological molecules and for the real-time identification of tissues in biological and clinical applications^3^. An attractive feature of MS-based tissue imaging is that it can be performed without using fluorescent probes or radioactive labels^3^. In addition, MS imaging (MSI) is a highly sensitive, rapid, and multiplexed technology that enables the simultaneous identification of a broad range of complex biomolecules in cell and tissue samples. To date, secondary ion MS, matrix-assisted laser desorption/ionization (LDI) MS, laser ablation electrospray ionization MS, and desorption electrospray ionization MS have been the major techniques used for MSI^3^. Mass spectrometric analysis using nanomaterials and nanostructured substrates has become a popular technique because of the versatility of the nanomaterials and nanosubstrates^7^. Recently, nanoparticles, including those prepared from gold, silver, and carbon nanotubes, have been employed as matrixes for LDI-based MSI analyses to achieve higher spatial resolution, lower background noise, and higher sensitivity^2^. Therefore, analyses of the distributions of specific molecular makers through simple nanoparticle-assisted LDI (NALDI) are in high demand for tissue imaging. Nevertheless, the drawback of MSI coupled with NALDI is the lack of sufficient specificity.

In this study, we found that graphene oxide (GO) modified with Mucin1 (MUC1) binding aptamer–conjugated gold nanoparticles (Apt_MUC1_–Au NPs/GO) coupled with LDI-MS is a facile platform for the detection of tumor cells and for tissue imaging (Fig. 1). MUC1 is a large transmembrane glycoprotein (250–500 kDa) of the mucin family; it consists of a varying amplified repeat sequence of 20 amino acid residues that is rich in serine, threonine, and proline residues in its extracellular domain, a hydrophobic membrane-spanning domain of 31 amino acid residues, and a cytoplasmic tail comprising 69 amino acid residues^3^. The overexpression (>10-fold) of MUC1 is often associated with a variety of malignant tumors, including breast, gastric, colorectal, lung, prostate, ovarian, pancreatic, and bladder carcinomas^4^, making MUC1 an ideal target molecule for tumor labeling and chemotherapeutics. Apt_MUC1_ (for its sequence, see the Method section) binds to synthetic peptides (*K*_d_: ca. 0.1 nM) containing MUC1 tandem repeated sequences of the extracellular domain^5^. In addition, Apt_MUC1_ has been employed to recognize and bind to native MUC1 at the surfaces of MCF-7 breast cancer cells^6^. In this study, we used LDI-MS analysis to demonstrate that the nanocomposite Apt_MUC1_–Au NPs/GO binds selectively to MCF-7 cells. In addition, we employed Apt_MUC1_–Au NPs/GO in combination with LDI-MS, monitoring the Au cluster ions ([Au_*n*_]^+^; Fig. 1c), for tumor tissue imaging. We conclude that the binding of Apt–Au NPs/GO with tumor cells can be transduced to highly amplified Au cluster ion signals during LDI-MS analysis.

## Results and Discussion

### LDI-MS of Apt_MUC1_–Au NPs/GO

**Figure 1:**
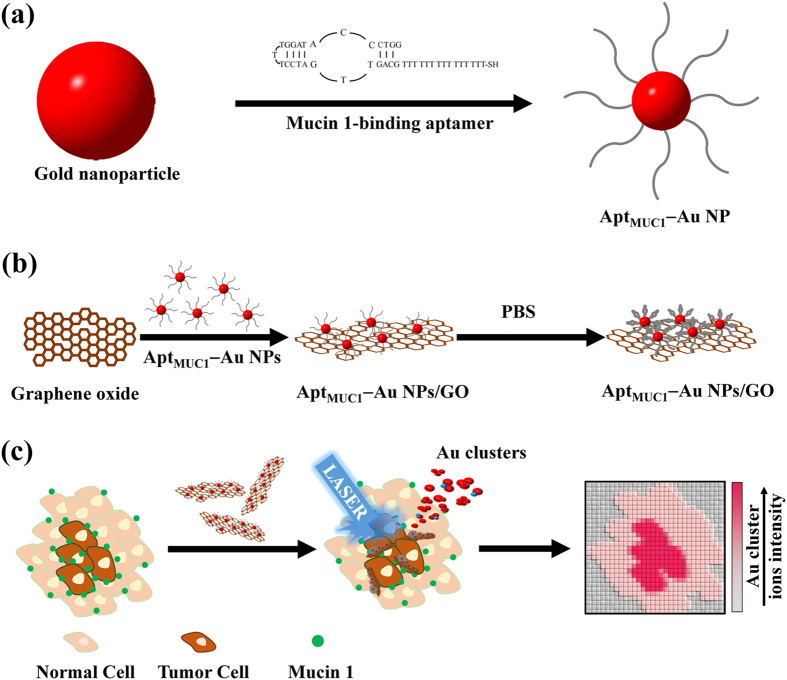
Schematic representation of (**a**) the preparation of MUC1-binding aptamer–modified gold nanoparticles (Apt_MUC1_–Au NPs) and (**b**) their conjugation to graphene oxide (Apt_MUC1_–Au NPs/GO) for (**c**) tumor tissue imaging by monitoring Au cluster ions when coupled with laser desorption/ionization mass spectrometry.

We synthesized GO from graphite (7–11 μm) using the modified Hummers method^7^. Atomic force microscopy (AFM) and transmission electron microscopy (TEM) images revealed that the average size of a single-layer GO (thickness: ca. 1.4 nm) was approximately 235 nm (see Supplementary Fig. S1a and S1b). The 5´-thiolated Apt_MUC1_, which comprised the functionality of a MUC1-binding aptamer (25-mer) and a polythymidine (T_15_) linker, was bound to the surfaces of the Au NPs (diameter: ca. 13 nm) through Au–S bonding, resulting in the preparation of Apt_MUC1_−Au NPs (ca. 50 Apt_MUC1_ molecules per Au NP). The 25-mer Apt_MUC1_ contains three Watson–Crick pairs, three consecutive mispairs, and four Watson–Crick pairs capped by a TTT triloop motif (Fig. 1a); their base-paired nucleotides melt cooperatively at approximately 47 °C. The TTT triloop structure is the primary binding site for MUC1 protein^5^. Apt_MUC1_–Au NPs/GO was prepared through simple mixing of Apt_MUC1_–Au NPs (1.0 nM) with GO (0.25 g L^–1^) in 5 mM sodium phosphate (pH 7.4). Apt_MUC1_–Au NPs were absorbed on GO with stabilization mainly through multivalent nucleobase–graphene π–π stacking between the linear Apt_MUC1_ and GO (Fig. 1b), although hydrophobic and hydrogen bonding interactions between Apt_MUC1_ and GO could not be excluded^9^. The conformation of Apt_MUC1_ was predominantly a stretched linear structure on the Au NP surfaces in a solution of low ionic strength (5 mM sodium phosphate, pH 7.4). Linear single-stranded DNA interacts with graphene more strongly than do hairpin and double-stranded DNA^1^. After the Apt_MUC1_–Au NPs had self-assembled on GO, we added phosphate-buffered saline (PBS; 10 mM sodium phosphate, 140 mM NaCl, 2.70 mM KCl; pH 7.6) to the Apt_MUC1_–Au NPs/GO solution to induce folding of the Apt_MUC1_ units into hairpin structures on the Au NP surfaces. As a result, the Apt_MUC1_ molecules could be arranged in an appropriate orientation and conformation for the binding of MUC1.

**Figure 2:**
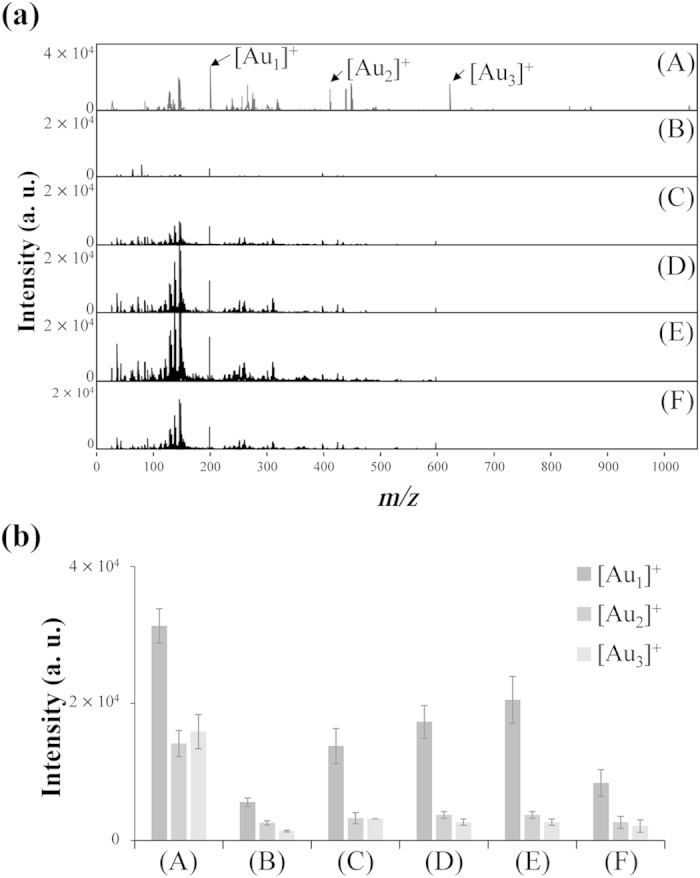
(**a**) LDI-MS spectra of (**A**) Au NPs (1.0 nM) and Apt_MUC1_–Au NPs (1.0 nM) in the (**B**) absence and (**C**–**F**) presence of GO at concentrations of (**C**) 0.0025, (**D**) 0.025, (**E**) 0.25, and (**F**) 2.5 g L^–1^. (**b**) Intensity of [Au_*n*_]^+^ signals obtained, under LDI, from (**A**) Au NPs (1.0 nM) and Apt_MUC1_–Au NPs (1.0030 nM) in the (**B**) absence and (**C**–**F**) presence of GO at concentrations of (**C**) 0.0025, (**D**) 0.025, (**E**) 0.25, and (**F**) 2.5 g L^–1^. Signals at *m*/*z* 196.97, 393.93, and 590.90 are assigned to [Au_1_]^+^, [Au_2_]^+^, and [Au_3_]^+^ ions, respectively. A total of 500 pulsed laser shots was applied to accumulate the signals from five LDI-targeted positions at a laser power density of 2.84 × 10^4^ W cm^−2^. Peak intensities are plotted in arbitrary units (a. u.). The error bars in (**b**) represent the standard deviations of five repeated measurements.

TEM imaging of Apt_MUC1_–Au NPs/GO revealed that the Apt–Au NPs had assembled homogeneously on the GO (see Supplementary Fig. S1c). UV–Vis absorption and DLS measurements (see Supplementary Fig. S2) revealed that Apt_MUC1_–Au NPs/GO dispersed well (no aggregation) in PBS solution. We used LDI to further study the formation efficiency of Au cluster ions from Apt_MUC1_–Au NPs and Apt_MUC1_–Au NPs/GO (Fig. 2). The photoabsorption of the Au NPs (13 nm) induced the desorption and ionization of the surface Au atoms under SmartBeam laser irradiation (355-nm Nd:YAG; 100 Hz; pulse width: 6 ns; 2.84 × 10^4^ W cm^–2^; beam diameter: ca. 40 μm). Photothermal evaporation and Coulomb explosion are two well-established mechanisms for the fragmentation of metallic NPs under pulsed laser illumination^2^. The Coulomb explosion mechanism involves the ejection of a large number of electrons to generate multiple ionized metallic clusters/nanoparticles^3^. In contrast, the photothermal mechanism suggests that the degree of surface evaporation is highly determined by laser-induced thermal energy transfer into the lattice system^3^. The formation of Au cluster ions from Au NPs under pulsed laser irradiation is very sensitive to the composition, size, and surface properties of the particles and the wavelength, intensity, and pulse width of the laser^5^. Similar to our previous findings^5^, the formation efficiency of the Au cluster ions [Au_*n*_]^+^ (*n*  = 1–3) from the Au NPs decreased to 16.7% after the Au NPs had been modified with Apt_MUC1_ (Figs. 2A, B). The Au NPs may have transferred their absorbed energy to the surface Apt_MUC1_ molecules, decreasing the lattice temperature (*T*_l_) of the Au NPs, after laser excitation^5^. As a result, the evaporation or ionization of the surface Au atoms/ions into the gas phase was suppressed. In addition, the dense mantle of Apt_MUC1_ units may have strongly inhibited electron ejection from the Au NPs, decreasing the electron temperature (*T*_e_) of the Au NPs; therefore, the explosive fragmentation-induced formation of Au cluster ions was strongly inhibited^3^.

We found that the formation of Au cluster ions from Apt_MUC1_–Au NPs was enhanced in the presence of GO. As revealed in Fig. 2B–F, the intensities of the Au cluster ions in the LDI-MS spectra increased upon increasing the concentration of GO (0–0.25 g L^−1^). Because of their high specific surface areas (ca. 2600 m^2^ g^–1^), high UV-region absorption coefficients, and energy transduction capabilities, graphene and GO have been used recently as nanomaterials in surface-assisted LDI-MS for the analysis of oligonucleotides, proteins, lipids, and small molecules^0^. We suspect that energy (heat) transfer from GO to the Apt_MUC1_–Au NPs may have been the main reason for the enhanced formation of [Au_*n*_]^+^ cluster ions; GO-assisted electron ejection from the Apt_MUC1_–Au NPs may also have contribute to the higher formation efficiency of the [Au_*n*_]^+^ cluster ions. The aggregation of Apt_MUC1_–Au NPs (1.0 nM; ~0.014 g L^–1^)/GO resulted in a slight decrease in the signal intensity of [Au_*n*_]^+^ when the concentration of GO was greater than 0.25 g L^–1^ (Fig. 2F). Aggregation occurred when the GO concentration was >0.25 g/L presumably because of the relative low number of adsorbed Apt_MUC1_–Au NPs on each GO and therefore, their stability in PBS solution decreased (see Supplementary Fig. S3 and Fig. S4). In addition, a low ratio (<0.06) of the concentrations of Apt_MUC1_–Au NPs (1.0 nM; ~0.014 g L^–1^) to GO (>0.25 g L^–1^) may have resulted in the formation of crosslinks (Apt_MUC1_–Au NPs/GO), thereby inducing aggregation of the nanocomposites. Such aggregation of Apt_MUC1_–Au NPs/GO nanocomposites made it difficult to fragment, desorb, and ionize the surface Au atoms/ions on the Au NPs. The combination of Apt_MUC1_–Au NPs/GO composites and LDI-MS monitoring of the [Au_1_]^+^ signals allowed detection of the Apt_MUC1_–Au NPs at concentrations as low as 10 fM. In contrast, the equivalent concentration from the UV–Vis absorption measurements, monitoring the surface plasmon resonance (SPR) absorption of the Au NPs at 520 nm, was approximately 10 pM (see Supplementary Fig. S5). In addition, the Au ion–driven signals arising from the high surface ratio of Au atoms/ions on Au NPs are much stronger than those from proteins in the LDI-MS analysis because of the low ionization/desorption efficiencies of proteins. Therefore, we suspected that our Apt_MUC1_–Au NPs/GO would be an exceptional labeling agent for the analysis of marker proteins in tumor cells when coupled with LDI-MS.

### Detection of tumor cells by Apt_MUC1_–Au NPs/GO–LDI-MS

**Figure 3:**
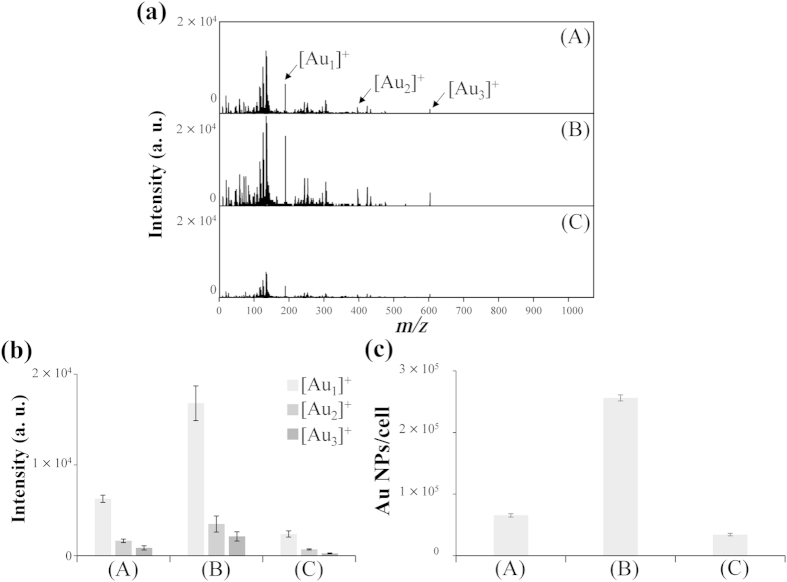
(**a**) LDI-MS spectra recorded using (**A**) Apt_MUC1_–Au NPs (1.0 nM), (**B**) Apt_MUC1_–Au NPs/GO ([Apt_MUC1_–Au NPs] = 1.0 nM; [GO] = 0.25 g L^–1^), and (**C**) T_45_–Au NPs/GO [(T_45_–Au NPs) = 1.0 nM; (GO) = 0.25 g L^–1^] a probes for the detection of 10^5^ MCF-7 cells. (**b**) Peak intensities of [Au_*n*_]^+^ ions obtained from (**A**) Apt_MUC1_–Au NPs, (**B**) Apt_MUC1_–Au NPs/GO, and (**C**) T_45_–Au NPs/GO after labeling of MCF-7 cells. (**c**) Concentrations of Au NPs accumulated in MCF-7 cells, as determined using ICP-MS. Other conditions were the same as those described in Fig. 1.

As revealed in Fig. 3a, b, the [Au_*n*_]^+^ signals obtained using Apt_MUC1_–Au NPs/GO, prepared from Apt_MUC1_–Au NPs (1.0 nM) and GO (0.25 g L^–1^), when coupled with LDI-MS for the detection of MUC1-overexpressing MCF-7 cells were approximately three times stronger than those obtained using Apt_MUC1_–Au NPs. In a control experiment, polythymine (T_45_)-conjugated Au NPs provided low-intensity [Au_*n*_]^+^ signals, compared with those from Apt_MUC1_–Au NPs and Apt_MUC1_–Au NPs/GO, when analyzing MCF-7 cells. The detection of MCF-7 cells using Apt_MUC1_–Au NPs/GO was superior to that using Apt_MUC1_–Au NPs, presumably because the former displayed a higher formation efficiency of Au cluster ions and/or stronger binding affinity toward MUC1 on the cell membrane. In a quantitation analysis performed using inductively coupled plasma mass spectrometry (ICP-MS), we found that the number of Au NPs bound or uptaken by MCF-7 cells from Apt_MUC1_–Au NPs/GO was approximately four times higher than that from Apt_MUC1_–Au NPs (Fig. 3c), revealing that Apt_MUC1_–Au NPs/GO had relatively stronger binding affinity toward MUC1 on the cell membrane. The signal enhancement obtained by LDI-MS (~3-fold) was lower than that of by ICP-MS (~4-fold) probably due to the incomplete fragmentation, desorption, and ionization of Apt_MUC1_−Au NPs to Au cluster ions in the LDI-MS analysis. The ultrahigh density of Apt_MUC1_ on the surface of the Au NPs and the ultrahigh density of Apt_MUC1_–Au NPs on the GO surface provided a high local concentration of flexible Apt_MUC1_ ligands, enhancing the cooperative and multivalent binding affinity toward MUC1 units on the cell membrane^2^. The nanosheet structure of GO, allowing ready adhesion to the plasma membrane and high uptake by mammalian cells through phagocytosis and clathrin-mediated endocytosisi^5^, may have contributed to the strong affinity of Apt_MUC1_–Au NPs/GO toward MCF-7 cells. In addition, high density of Au NPs on GO can generate greater number of Au cluster ions for signal enhancement. Our Apt_MUC1_–Au NPs/GO–LDI-MS system could detect as few as 100 MCF-7 cells when monitoring the [Au_1_]^+^ signals (see Supplementary Fig. S6). Compared to other mass spectrometry based tumor cell analysis techniques^8^, our Apt_MUC1_–Au NPs/GO–LDI-MS platform is relatively simple, rapid, and selective and shows great legibility for tumor cells quantification.

### Specificity of Apt_MUC1_–Au NPs/GO

We further applied our Apt_MUC1_–Au NPs/GO–LDI-MS platform to analyze other types of cells, namely MCF-10A (normal mammary epithelial cells), MDA-MB-231 (breast adenocarcinoma cells), and 293T (transformed embryonic kidney cells). Among the four tested cell lines, the highest-intensity [Au_1_]^+^ signals arose from the MCF-7 breast cancer cell line (see Supplementary Fig. S7a and S7b), consistent with our results from ICP-MS analysis (see Supplementary Fig. S7c). Notably, the sample preparation for ICP-MS analysis required relatively complicated and tedious pretreatment steps. We also observed that the intensity of the [Au_1_]^+^ signals increased upon increasing the ratio of MCF-7 to MCF-10A cells in a co-culture system containing these two cell types (see Supplementary Fig. S8). MUC1 is widely expressed by normal epithelial cells (e.g., MCF-10A); this expression increases significantly when the cells become malignant, such as in breast, pancreatic, or ovarian cancer^4^. MUC1 is overexpressed in approximately 90% of human breast cancers; the expression of membrane-associated MUC1 in MDA-MB-231 cells is, however, lower than that in MCF-7 cells^9^. The low level of MUC1 expression in some breast adenocarcinoma cells is associated with decreased cytokeratin expression and increased vimentin expression^0^. In fact, the MUC1 protein is expressed aberrantly in many solid tumor cell lines^1^. For example, the 293T cell line is MUC1-negative because it does not expresses endogenous MUC1^1^. Our results from Apt_MUC1_–Au NPs/GO–LDI-MS analysis platform (Fig. S7) are consistent with these reports and the results of the western blotting analysis (see Supplementary Fig. S9)^1^. MUC1 is associated with cellular transformation and tumorigenicity; it is considered as both a potential cancer biomarker and an attractive target for cancer immunotherapy^4^. Therefore, we suspect that our proposed assay will have practical applications in the detection of MUC1 expression in tumor cells, offering high accuracy and reliability with comprehensive information for the early detection of MUC1-related cancers.

### Analysis of MUC1 expression

**Figure 4:**
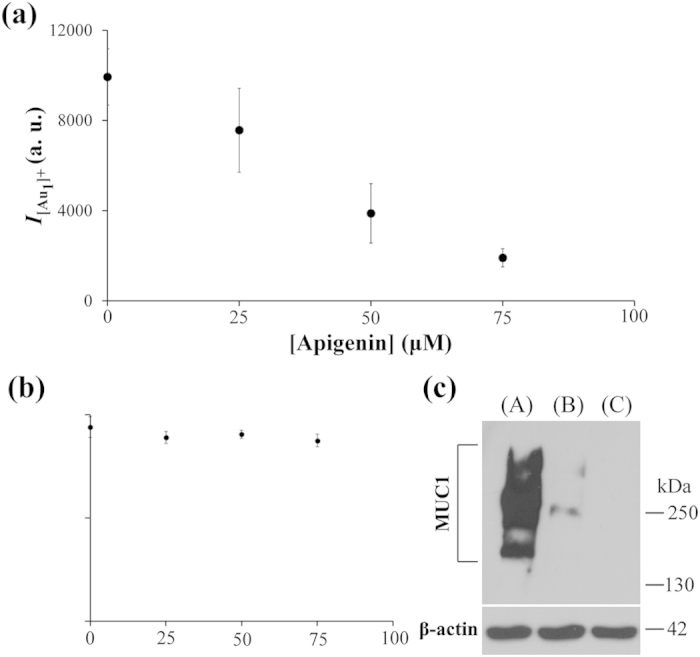
(**a**) Mass signal intensities of [Au^+^] ions (*I*_[Au1]+_) obtained when using Apt_MUC1_–Au NPs/GO to analyze apigenin-treated (0–75 μM) MCF-7 cells (10^5^ cells well^–1^). (**b**) Cell viability of MCF-7 cells incubated with apigenin in alpha-MEM at 37 °C under 5% CO_2_ for 24 h. (**c**) Western blotting for MUC1 in the cell lysates of (**A**) MCF-7 cells (10^5^ cells well^–1^), (**B**) MCF-7 cells (10^5^ cells well^–1^) after treatment with 75 μM apigenin for 24 h, and (**C**) 293T cells (10^5^ cells well^–1^). Other conditions were the same as those described in Fig. 2.

Furthermore, we applied our Apt_MUC1_–Au NPs/GO–LDI-MS platform to the analysis of MCF-7 cells after treatment with apigenin (4´,5,7-trihydroxyflavone; 0–75 μM) for 24 h. Apigenin, a natural polyphenol product belonging to the flavone class, is abundant in some fruits (e.g., cherries, apples, grapes) and vegetables (e.g., parsley, Chinese cabbage, bell peppers, celery). Apigenin has been studied widely for its anti-inflammatory, antioxidant, and antitumor properties; it can inhibit tumor cell invasion and metastases by regulating protease production^2^. Recently, it was demonstrated that apigenin can inhibit mucin gene expression and the production of mucin protein in epithelial and tumor cells^3^. Using our Apt_MUC1_–Au NPs/GO–LDI-MS detection system, we also observed an apigenin-induced downregulation of MUC1 expression: the intensity of the [Au_1_]^+^ signals decreased upon increasing the concentration of apigenin used for treatment of the MCF-7 cells (Fig. 4a). This downregulation of MUC1 in MCF-7 cells was consistent with the results of western blotting (Fig. 4c), revealing that our LDI-MS–based assay has great potential for use in the analysis of membrane protein expression levels after labeling with functional Au NPs. Compared with western blotting analysis, our assay is relatively simple, sensitive, and capable of high throughput.

### Tissue imaging

The development of a rapid, high-throughput method for MUC1 expression imaging in a typical tissue microarray would be a significant advance over the current practice. Therefore, we further employed our Apt_MUC1_–Au NPs/GO–LDI-MS platform for tissue microarray imaging by monitoring the [Au_1_]^+^ signals after the Apt_MUC1_–Au NPs had targeted cells in a tissue microarray (US Biomax). The tissue microarray, comprising breast carcinoma or normal tissues (diameter: 1.5 mm) was labeled with Apt_MUC1_–Au NPs/GO and subsequently studied using LDI-MS. Representative images for breast carcinoma and normal microarray tissues sections (see Supplementary Fig. S10) revealed that the total intensity of the [Au_1_]^+^ signals from the breast carcinoma tissue was much higher (>10-fold) than that from the normal tissue, suggesting that our Apt_MUC1_–Au NPs/GO–LDI-MS system is capable of analyzing MUC1 expression in tissue samples. The spatial resolution of our LDI-MS imaging technique (~80 μm) is limited by laser beam diameter size (40 μm) which let the spatial resolution inferior to the current practices such as fluorescence imaging^4^. However, Apt_MUC1_–Au NPs/GO–LDI-MS platform provides the great specificity and sensitivity for the molecular imaging due to the specific and strong binding of Apt_MUC1_–Au NPs/GO to targeted molecules on cell membrane and the high signal enhancement from Au NPs.

**Figure 5:**
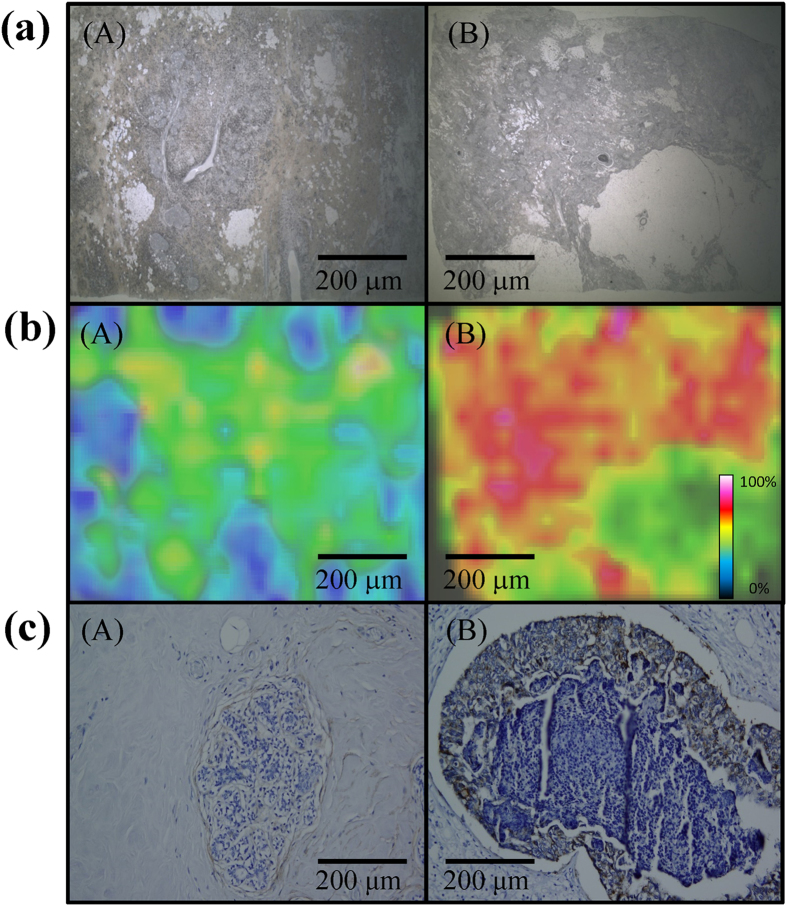
(**a**) Optical images of (**A**) normal breast and (**B**) breast tumor tissues. (**b**) LDI-MS images of the [Au_1_]^+^ intensity distributions in (**A**) normal breast and (**B**) breast tumor tissues after incubation with Apt_MUC1_–Au NPs/GO for 1 h. (**c**) Immunostaining of MUC1 by MUC1 antibody (VU4H5) in (**A**) normal breast and (**B**) tumor breast tissues. The dark brown and blue colors represent the staining of MUC1 and nuclei, respectively. Parameters of LDI-MS imaging: image pixel size, 1 × 1 μm^2^; line distance, 50 μm; repetition rate, 100 Hz; image size, 0.8 × 0.8 mm^2^. Other conditions were the same as those described in Fig. 3.

We also applied our Apt_MUC1_–Au NPs/GO–LDI-MS platform to analyze the tissue sections (US Biomax) of normal breast tissue (normal human mammary gland; female, 35 years old) and human breast cancer tissue (human breast invasive ductal carcinoma; female, 42 years old). As indicated in Fig. 5b, MSI revealed, through monitoring of the intensity of [Au_1_]^+^ signals, a dramatic difference between the normal and tumor tissues, suggesting high sensitivity of our LDI-MS–based tumor tissue system for the diagnosis of malignancies. We used immunohistochemistry (IHC) to further validate MUC1 expression and distribution in tumor and normal breast tissue. Figure 5c presents the immunostaining images of the sections; the results are in good agreement with those obtained through LDI-MS, with the heterogeneously developed brown color being deeper in the breast tumor tissue (Fig. 5cB). These IHC validation studies confirmed that the expression pattern of MUC1 reflects the tissue distribution in the malignant breast cells.

In summary, we have coupled an Apt_MUC1_–Au NPs/GO probe with LDI-MS for the detection of breast cancer cells and for tissue imaging. The formation efficiency of [Au_*n*_]^+^ ions from Apt_MUC1_–Au NPs under pulsed laser irradiation was enhanced after anchoring to GO. In addition, Apt_MUC1_–Au NPs/GO could bind specifically to MUC1 units on cell membranes. The intensities of the signals for [Au_*n*_]^+^ cluster ions in the mass spectra revealed the levels of MUC1 expression in tumor cells; these signals behaved as a highly amplified target-labeling indicator for the targeted tumor cells. Our probe enabled the selective and sensitive detection of as few as 100 MCF-7 cells and facilitated efficient analyses of MUC1 protein expression in four different cell lines. We also applied the Apt_MUC1_–Au NPs/GO probe to breast tumor tissue imaging, highlighting the potential for studying various cancer tissues with different aptamer-modified Au NPs/GO. To the best of our knowledge, this study provides the first example of combining Apt_MUC1_–Au NPs/GO with LDI-MS for tumor tissue imaging. This high-throughput LDI-MS imaging system has great potential for the diagnosis of cancer.

## Methods

### Materials and reagents

All thiolated oligonucleotides used in this study were purchased from Integrated DNA Technologies (Coralville, IA). Graphite powder (99%, 7–11 μM) was purchased from Alfa–Aesar (Heysham, Lancashire, UK). Sulfuric acid, phosphoric acid, hydrogen peroxide, trisodium citrate, sodium phosphate tribasic, sodium phosphate dibasic, boric acid, tetrachloroauric acid, dimethyl sulfoxide, apigenin, xylene, ethanol, 3,3-diaminobenzidine tetrahydrochloride, hematoxylin, and isopropanol were purchased from Sigma–Aldrich (Milwaukee, WI). Citric acid, calcium chloride, magnesium chloride, potassium permanganate, tris(hydroxymethyl)aminomethane (Tris), and hydrochloric acid were purchased from Mallinckrodt Baker (Phillipsburg, NJ). Alpha-MEM and fetal bovine serum were purchased from GIBCO (Campinas, Brazil). An MTT assay kit was purchased from Thermo Fisher Scientific (Waltham, MA). MUC1 (VU4H5) mouse mAb, anti-mouse IgG, and HRP-linked antibody were purchased from Cell Signaling Technology (Danvers, MA). Milli-Q ultrapure water (18 MΩ, Millipore, Billerica, MA) was used in all experiments.

### Preparation of Apt_MUC1_–Au NPs/GO

A thiol-modified MUC1-binding aptamer (5´-HS-TTT TTT TTT TTT TTT GCA GTT GAT CCT TTG GAT ACC CTG G-3´) was attached to the Au NPs following a slightly modified procedure reported elsewhere^5^. An aliquot of aqueous Au NP solution (980 μL) in a 1.5-mL tube was mixed with the thiol-oligonucleotide (100 μM, 20 μL) to yield final concentrations of 15 nM Au NPs and 2 μM oligonucleotides. After 2 h, the mixed solution was salt-aged in the presence of 200 mM NaCl for over 16 h. Salt-aging resulted in a greater number of thiol-modified oligonucleotides bound to the Au NPs^5^. The mixture was then centrifuged at an relative centrifugal force (RCF) of 30,000 × *g* for 20 min to remove the excess thiol-oligonucleotide. After removal of the supernatant, the oily precipitate was washed with 5.0 mM Tris–HCl (pH 7.6). After three centrifuge/wash cycles, the colloid was resuspended in 5.0 mM Tris–HCl (pH 7.6) and stored at 4 °C. The Apt_MUC1_–Au NPs/GO nanocomposite was prepared by mixing the GO (0.25 g L^–1^) and Apt_MUC1_–Au NPs (1.0 nM) in 5 mM sodium phosphate (pH 7.4). After incubation for 1 h, the Apt_MUC1_–Au NPs/GO was transferred into PBS and incubated for another 1 h at room temperature.

### Analysis of cells by Apt_MUC1_–Au NPs/GO–LDI-MS

Cells were cultured on 8-mm chips in 48-well plates for 8 h at 37 °C. For co-culture experiments, MCF-10A/MCF-7 cells were mixed and seeded at different ratios. The cultured cells were labeled separately with Apt_MUC1_–Au NPs/GO for 1 h in physiological buffer. The cell adhesive chips were then moved out and washed three times with PBS. The chips were then cast onto a stainless-steel 384-well MALDI target and air-dried at room temperature prior to LDI time-of-flight (TOF) MS measurements. MS experiments were performed in the reflectron positive-ion mode using an Autoflex III MALDI TOF/TOF mass spectrometer (Bruker Daltonics, Bremen, Germany). The samples were irradiated using a SmartBeam laser (355 nm Nd: YAG) operated at 100 Hz. Ions produced by LDI were energetically stabilized during a delayed extraction period of 30 ns and accelerated through the TOF chamber in the reflection mode prior to entering the mass-analyzer. The available accelerating voltages ranged from +20 to –20 kV. The instruments were calibrated with Au clusters, using their theoretical masses, prior to analysis. A total of 500 pulsed laser shots were applied to accumulate signals from five MALDI target positions at a power density of 2.84 × 10^4^ W cm^–2^. For ICP-MS (Agilent 7700 Series, Agilent Technologies, Santa Clara, CA), the Apt_MUC1_–Au NPs/GO-treated cells samples were prepared in 2% HNO_3_.

### Tissue imaging

The human breast tumor and normal breast tissue microarray (BR811; 80 cases; diameter: 1.5 mm; thickness: 5 μm), human normal breast tissue section (HuFPT130), and human breast cancer section (HuCAT298) were used for tissue imaging. The procedures of tissue sample treatments were performed in accordance with the protocols approved by Animal Administration Committee of National Taiwan Ocean University. After deparaffinization, the tissue samples were labeled with Apt_MUC1_–Au NPs/GO, prepared from 1.0 nM Apt_MUC1_–Au NPs and 0.25 g L^–1^ GO, in PBS for 1 h. The slides were washed three times with PBS before they were cast onto an MTP slide adapter II MALDI plate and air-dried at room temperature prior to LDI-MS measurements. LDI-MS imaging experiments were performed in the image reflectron positive-ion mode using an Autoflex III MALDI TOF/TOF mass spectrometer. The tissue slides were irradiated using a SmartBeam laser (355 nm Nd: YAG) operated at 100 Hz. A laser spot diameter of 40 μm and a raster width of 50 μm were employed. Ions produced by LDI were energetically stabilized during a delayed extraction period of 30 ns and accelerated through the TOF chamber in the reflection mode prior to entering the mass-analyzer. The available accelerating voltages ranged from +20 to –20 kV. The instruments were calibrated with Au clusters, using their theoretical masses, prior to analysis. One spot was pulsed for a total of 500 laser shots at a power density of 2.84 × 10^4^ W cm^−2^.

Please see the Supplementary Information for the details on the preparation and characterization of GO, preparation of 13-nm spherical Au NPs, cell cultures, and immunohistochemistry with horseradish peroxidase.

## Author Contributions

R.C.H. and C.C.H designed the research. R.C.H. performed most of the experiments and data analysis. W.J.C. contributed to the characterization of the nanocomposites. I.P.J.L. contributed to western blotting analysis. R.C.H. and C.C.H co-wrote the paper. All authors discussed the results and commented on the manuscript.

## Additional Information

**How to cite this article**: Huang, R.-C. *et al.* Multivalent Aptamer/Gold Nanoparticle-Modified Graphene Oxide for Mass Spectrometry-Based Tumor Tissue Imaging. *Sci. Rep.* **5**, 10292; doi: 10.1038/srep10292 (2015).

## Supplementary Material

Supplementary Information
