# Supplementary material for: Multivalent Aptamer/Gold Nanoparticle–Modified Graphene Oxide for Mass Spectrometry–Based Tumor Tissue Imaging
Source: Sci Rep. 2015 May 14;5:10292. doi: 10.1038/srep10292 (PMC4431351; doi:10.1038/srep10292)
Supplement: Supplementary Information [file srep10292-s1.doc]

***Supplementary Information***

**Multivalent Aptamer/Gold Nanoparticle–Modified Graphene Oxide for Mass Spectrometry–Based Tumor Tissue Imaging**

Rong-Cing Huang^1^, Wei-Jane Chiu^1^, Irving Po-Jung Lai^1^ and Chih-Ching Huang^1,2,3^,*

^1^Department of Bioscience and Biotechnology, National Taiwan Ocean University, Keelung, 20224, Taiwan; ^2^Center of Excellence for the Oceans, National Taiwan Ocean University, Keelung, 20224, Taiwan; ^3^School of Pharmacy, College of Pharmacy, Kaohsiung Medical University, Kaohsiung, 80708, Taiwan

**Correspondence:** Chih-Ching Huang, Department of Bioscience and Biotechnology, National Taiwan Ocean University, 2, Pei-Ning Road, Keelung 20224, Taiwan; Tel.: 011-886-2-2462-2192 ext. 5517; Fax: 011-886-2-2462-2320; E-mail: [huanging@ntou.edu.tw](mailto:huanging@ntou.edu.tw)

**EXPERIMENTAL**

**Preparation and characterization of GO**

GO was prepared using a modification of the Hummers method.^r1^ In brief, a mixture of concentrated H_2_SO_4_ (360 mL) and concentrated H_3_PO_4_ (40 mL) was added to a mixture of graphite powder (3.0 g) and KMnO_4_ (18.0 g). The resulting mixture was heated at 50 ℃, stirred for 12 h, and then cooled to room temperature in ice and poured onto DI water (100 mL) containing 30% H_2_O_2_ (3 mL). After centrifuging at a relative centrifugal force (RCF) of 35,000 × *g* for 1 h, the supernatant was decanted. The remaining pellet was repeatedly washed with DI water (200 mL) until pH 6 was reached. After sonication for 1 h, the GO solution was centrifuged at a RCF of 25,000 × *g* for 0.5 h to remove the aggregated GO. The concentration of GO in the supernatant, determined using the freeze dry method, was approximately 2.5 g L^–1^; this sample is denoted herein as 100X. Dynamic light scattering (DLS) and zeta potential experiments were performed on GO using a Zetasizer 3000 HS analyzer (Malvern Instruments, Malvern, UK). Transmission electron microscopy (TEM) was performed using an HT-7700 system (Hitachi, USA), operated at 75 kV.

**Preparation of 13-nm spherical Au NPs**

Aqueous 4.0 mM trisodium citrate (50 mL) was brought to a vigorous boil while stirring in a round-bottom flask fitted with a reflux condenser. HAuCl_4_ (1.0 mM, 0.5 mL) was added rapidly and then the mixture was heated for another 8 min, during which time the color changed from pale yellow to deep red. The solution was cooled to room temperature with continuous stirring. TEM revealed nearly monodisperse Au NPs having an average size of 13.3 ± 1.2 nm. A double-beam UV–Vis spectrophotometer (Cintra 10e, GBC, Victoria, Australia) was used to measure the absorption of the Au NP solution. The particle concentration of the Au NPs (15 nM) was determined according to Beer’s law using an extinction coefficient of 2.43 × 10^8^ M^–1^ cm^–1^ at 520 nm for 13.3-nm Au NPs.^r2^

**Cell culture**

Breast tumor cell lines (MDA-MB-231 and MCF-7), immortalized normal mammary epithelial cell line (MCF-10A), and transformed human embryonic kidney cell line (293T) were purchased from the American Type Culture Collection (Manassas, VA). In brief, the cells were maintained in alpha-MEM containing 10% fetal bovine serum. MCF-10A cells were cultured in alpha-MEM supplemented with prequalified human recombinant epidermal growth factor 1-53 (EGF 1-53; Invitrogen, Carlsbad, CA) and bovine pituitary extract (Invitrogen). All cells were cultured in a humidified incubator at 37 °C under 5% CO_2_. The MCF-7 cells were cultured in the presence of different concentrations of apigenin to inhibit MUC1 expression. Following the separated incubation of MCF-7 (ca. 10^6^ cells mL^–1^; 0.5 mL well^–1^) in a culture medium for 24 h at 37 °C under 5% CO_2_, the culture medium was replaced with 0.5 mL of the medium containing apigenin at various concentrations (0–75 μM). The cells were then incubated for another 72 h. The cells were carefully rinsed three times with PBS, followed by treatment with Alamar Blue reagent (10-fold dilution, 0.5 mL well^–1^) for 4 h. The fluorescence arising from reduction of the dye by the live cells was measured using a fluorescence microplate reader (Synergy 4) from BioTek (Winooski, VT), with an excitation wavelength of 545 nm and an emission wavelength of 590 nm. Because the fluorescence was directly proportional to the cell quantity, it was used to calculate cell viability, assuming 100% viability in the control set (media containing no apigenin).

**Immunohistochemistry with horseradish peroxidase**

Human normal breast tissue sections (HuFPT130) and human breast tumor sections (HuCAT298) in formalin-fixed and paraffin-embedded condition were purchased from Biomax (Rockville, MD). First, the paraffin-embedded tissue sections required deparaffinization. The tissue slides were soaked in xylene (99%) for 10 min and then placed in fresh xylene and incubated for another 10 min; they were then immersed sequentially in 100, 95, and 70% EtOH (5 min each). Finally, the surfaces of the slides were cleaned with deionized water. The first antibody was MUC1 (VU4H5) mouse antibody; the second was anti-mouse IgG, HRP-linked antibody. The staining reagents were 3,3-diaminobenzidine tetrahydrochloride and hematoxylin. HRP oxidized 3,3-diaminobenzidine tetrahydrochloride to stain the location of the antibody in the form of dark brown-colored products. Hematoxylin was used to stain the nuclei with blue color.

**REFERENCES**

r1. Marcano, D. C. *et al.* Improved synthesis of graphene oxide. *ACS Nano* **4**, 4806–4814 (2010).

r2. Link S & El-Sayed, M. A. Spectral properties and relaxation dynamics of surface plasmon electronic oscillations in gold and silver nanodots and nanorods. *J. Phys. Chem. B* **103**, 8410–8426 (1999).


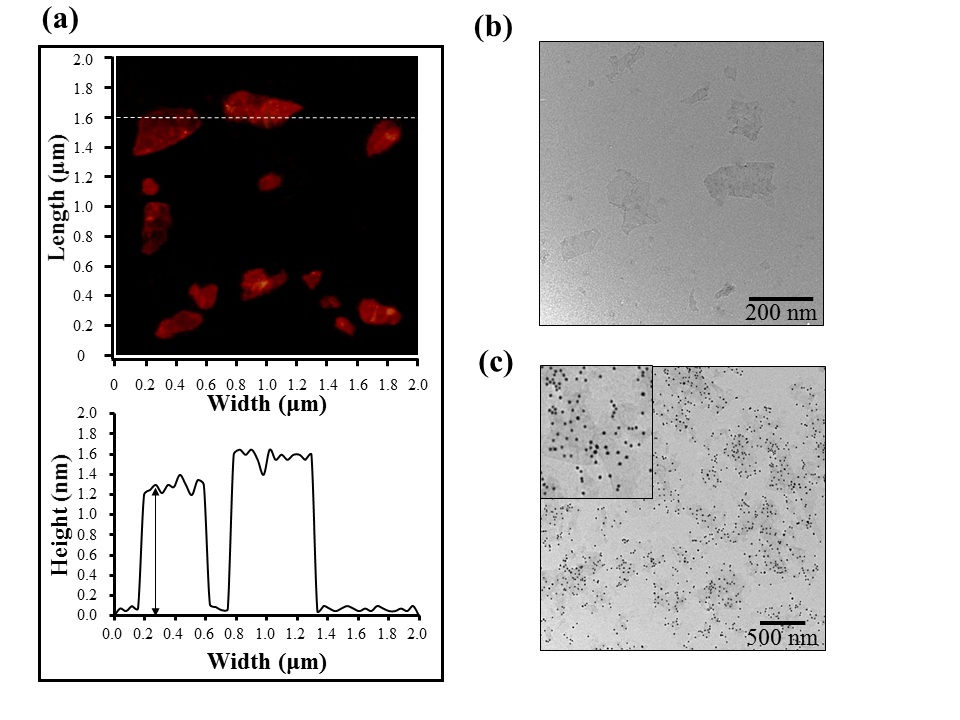


***Figure S1.*** (a) AFM and (b) TEM images of the as-synthesized GO. (c) TEM image of the as-prepared Apt_MUC1_–Au NPs/GO. Inset to Figure 1c: high magnification TEM image of the Apt_MUC1_–Au NPs/GO.

***Figure S2.*** (a) UV–Vis absorption spectra and (b) DLS traces of (A) Apt_MUC1_–Au NPs (1.0 nM) and (B) Apt_MUC1_–Au NPs/GO (prepared from 1.0 nM Apt_MUC1_–Au NPs and 0.25 g L^–1^ GO) in PBS solution and (C) GO (0.25 g L^–1^) in deionized water.

***Figure S3.*** TEM images of PBS solutions containing Apt_MUC1_–Au NPs (1.0 nM) in the presence of GO at concentrations of (a) 0.0025, (b) 0.025, (c) 0.25, and (d) 2.5 g L^–1^. Insets: Photographs of the corresponding Apt_MUC1_–Au NPs/GO solutions.


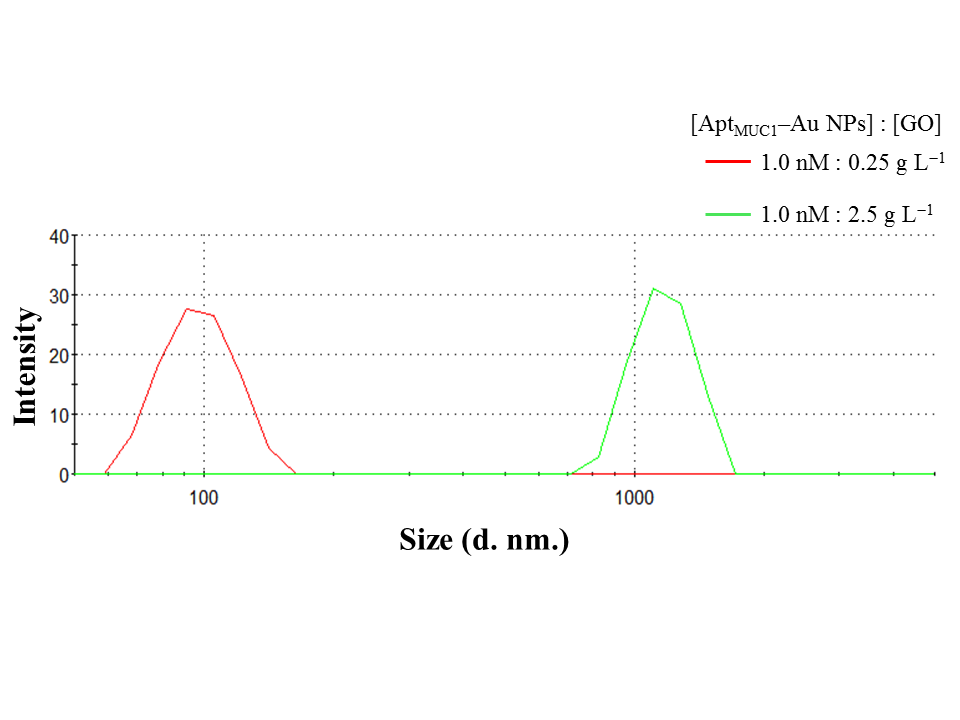


***Figure S4.*** DLS sizes of Apt_MUC1_–Au NPs (1.0 nM) in PBS solutions in the presence of GO at concentrations of 0.25 g L^–1^ (red curve) and 2.5 g L^–1^ (green curve). Other conditions were the same as those described in Figure S3. DLS measurements reveal that the sizes of the Apt_MUC1_–Au NPs (1.0 nM)/GO (0.25 g L^–1^) and Apt_MUC1_–Au NPs (1.0 nM)/GO (2.5 g L^–1^) were around 100 and 1050 nm, respectively, further confirming that the aggregation occurred when the GO concentration was >0.25 g/L.

***
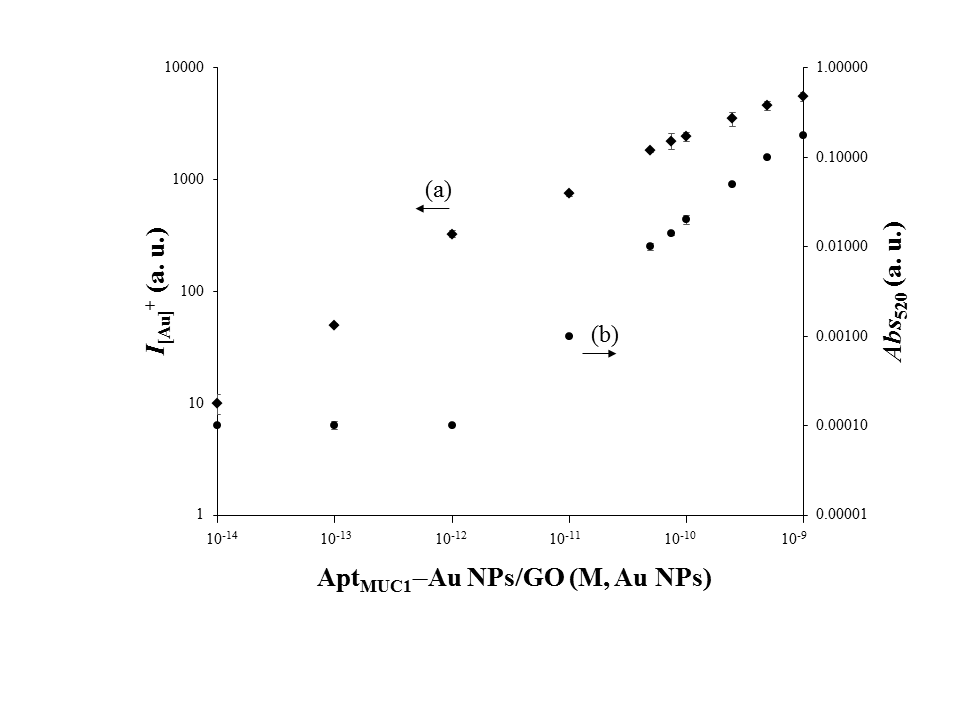
***

***Figure S5.*** Plots of (a) the [Au_1_]^+^ peak intensity (*I*_[Au]_^+^), recorded through LDI-MS, from Apt_MUC1_–Au NPs (10 fM–1.0 nM) and (b) the UV–Vis absorption at 520 nm (SPR band; *Abs*_520_) of Apt_MUC1_–Au NPs (10 fM–1.0 nM) in PBS solution. Error bars represent standard deviations from three repeated measurements.

***Figure S6.*** (a) LDI mass spectra recorded using Apt_MUC1_–Au NPs/GO as a probe for the detection of (A) 10, (B) 100, (C) 1000, (D) 10,000, and (E) 50,000 MCF-7 cells. (b) Plots of the [Au_1_]^+^ intensity with respect to the number of MCF-7 cells (10–50,000 cells). A total of 500 pulsed laser shots were applied to accumulate the signals from five LDI-targeted positions at a laser power density of 2.84 × 10^4^ W cm^−2^. Peak intensities in (a) and the intensities of the signals for [Au^+^] (*I*_[Au1]+_) in (b) are plotted in arbitrary units (a. u.). The error bars in (b) represent standard deviations from five repeated measurements. Other conditions were the same as those described in Figure S5.

***Figure S7.*** (a) Mass spectra recorded using Apt_MUC1_–Au NPs/GO as a probe for the analysis of (A) MCF-10A, (B) MCF-7, (C) MDA-MB-231, and (D) 293T cells (10^5^ cells well^–1^). (b) Peak intensities of [Au^+^] ions (*I*_[Au1]+_) obtained from Apt_MUC1_–Au NPs/GO-labeled (A) MCF-10A, (B) MCF-7, (C) MDA-MB-231, and (D) 293T cells. (c) Concentration of Au NPs accumulated in (A) MCF-10A, (B) MCF-7, (C) MDA-MB-231, and (D) 293T cells, as determined using ICP-MS. Error bars represent standard deviations from five repeated measurements. Other conditions were the same as those described in Figure S6.

***Figure S8.*** (a) Apt_MUC1_–Au NPs/GO–LDI-MS analyses of co-cultures of MCF-7 and MCF-10A cells at ratios of (A) 0 : 10, (B) 2 : 8, (C) 5 : 5, (D) 8 : 2, and (E) 10 : 0. (b) Peak intensities of [Au_1_]^+^ ions obtained after labeling co-cultures of MCF-7 and MCF-10A (various ratios) with Apt_MUC1_–Au NPs/GO. Other conditions were the same as those described in Figure S7.

***
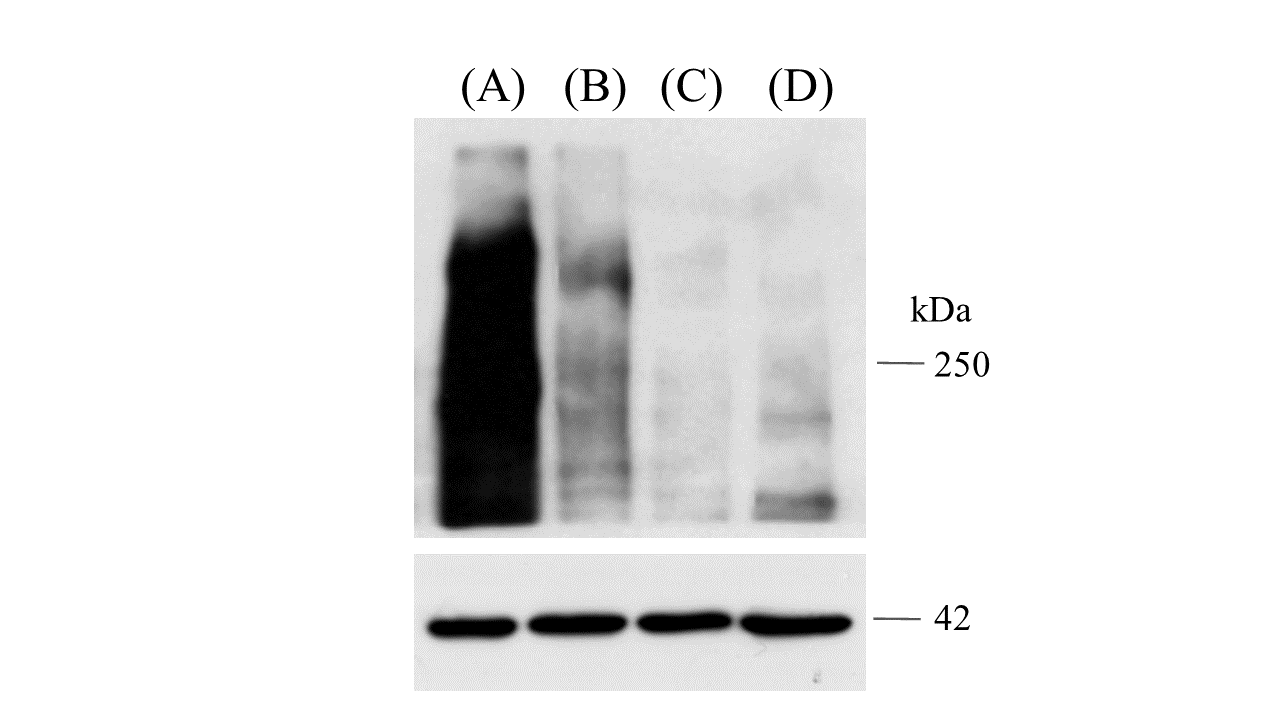
***

***Figure S9.*** Western blotting for MUC1 in the cell lysates of (A) MCF-7, (B) MCF-10A, (C) MDA-MB-231, and (D) 293T cells (10^5^ cells well^–1^). Other conditions were the same as those described in Figure 3c.

***Figure S10.*** (a) Optical images of (A) normal breast and (B) breast tumor microarray tissues. (b) LDI-MS images of the [Au_1_]^+^ intensity distributions in (A) normal breast and (B) breast tumor microarray tissues after labeling with Apt_MUC1_–Au NPs/GO. Other conditions were the same as those described in Figure 6.
